# Successful Endoscopic Excision for a Rapidly Enlarging Esophageal Histopathologically Unclassified Subepithelial Lesion: A Case Report

**DOI:** 10.1002/deo2.70219

**Published:** 2025-10-15

**Authors:** Mai Fukuda, Naoya Tada, Miku Maeda, Koichi Oishi, Mamoru Ito, Yuko Hasegawa, Toshiki Futakuchi, Masakuni Kobayashi, Naoto Tamai, Masayuki Shimoda, Kazuki Sumiyama

**Affiliations:** ^1^ Department of Endoscopy The Jikei University School of Medicine Tokyo Japan; ^2^ Digestive Diseases Center, Showa University Koto Toyosu Hospital Tokyo Japan; ^3^ Department of Pathology The Jikei University School of Medicine Tokyo Japan

**Keywords:** endoscopic submucosal dissection, esophageal subepithelial lesion, giant SEL, inflammatory changes, multiple biopsies

## Abstract

A 75‐year‐old man presented with an esophageal subepithelial lesion (SEL) measuring 2.5 cm, first identified over a decade ago. The patient was followed up regularly with computed tomography and endoscopy and remained asymptomatic since then. However, over the past year, the patient developed dysphagia, and endoscopic evaluation revealed that the tumor had enlarged to 6.0 cm. Although nine endoscopic examinations with biopsies were performed, no definitive histopathological diagnosis was established. Endoscopic ultrasonography revealed that the tumor originated primarily from the submucosa. Given the rapid growth of tumor size and progressive symptoms, the tumor was removed with endoscopic submucosal dissection (ESD) in an en bloc manner. Histopathological analysis revealed a SEL characterized by vascular proliferation, thickening of the lamina muscularis mucosa, and inflammatory changes. No evidence of neoplasm was identified, suggesting the presence of a reactive lesion. The patient's dysphagia improved following ESD, and no recurrence was observed during a 15‐month follow‐up period. To date, no reports have documented rapidly growing esophageal SELs with abundant vascularization during follow‐up.

## 1 Introduction

Esophageal subepithelial lesions (SELs) are often detected incidentally during esophagogastroduodenoscopy (EGD). The most common esophageal SEL is leiomyoma, accounting for 60%–70%, whereas gastrointestinal stromal tumors and granular cell tumors are less frequent [1, 2]. Consequently, most lesions without high‐risk features, such as ulceration, size increase, or symptom development, are managed conservatively. However, some SELs enlarge or become symptomatic, necessitating further evaluation to exclude malignancy. Definitive diagnosis can be challenging because endoscopic biopsy and imaging studies are often inconclusive. Endoscopic submucosal dissection (ESD), initially developed for en bloc removal of superficial gastrointestinal neoplasms, can also be used for SELs arising from the submucosa [3]. Herein, we report a rare case of an esophageal SEL that was initially small but demonstrated rapid enlargement and symptomatic progression during long‐term follow‐up, remaining histologically unclassified even after ESD.

### 1.1 Case Report

**FIGURE 1:**
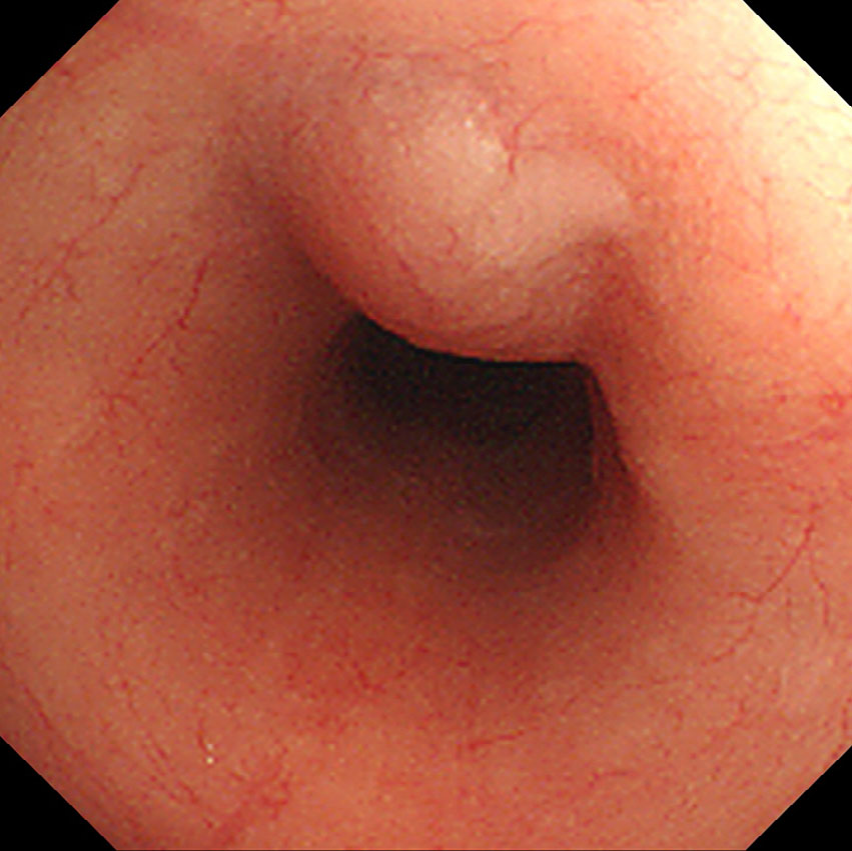
This is an image of a subepithelial lesion (SEL) from the initial esophagogastroduodenoscopy (EGD). A 25 mm yellowish‐white subepithelial lesion in the esophagus 4 years before the endoscopic resection.

A 75‐year‐old was initially found to have a 2.5 cm SEL in the esophagus 10 years ago. EGD at that time revealed a soft, elevated lesion with a smooth surface, normally colored, 28 cm from the incisors (middle thoracic esophagus), that demonstrated good mobility (Figure 1). Five years later, he developed pharyngeal and laryngeal edema of unknown etiology and underwent tracheostomy. Four years ago, he was noted to have multiple enlarged lymph nodes and was found to be Epstein‐Barr Virus (EBV)‐DNA positive (5.03IU/mL), raising suspicion for EBV‐associated lymphoproliferative disease.

**FIGURE 2:**
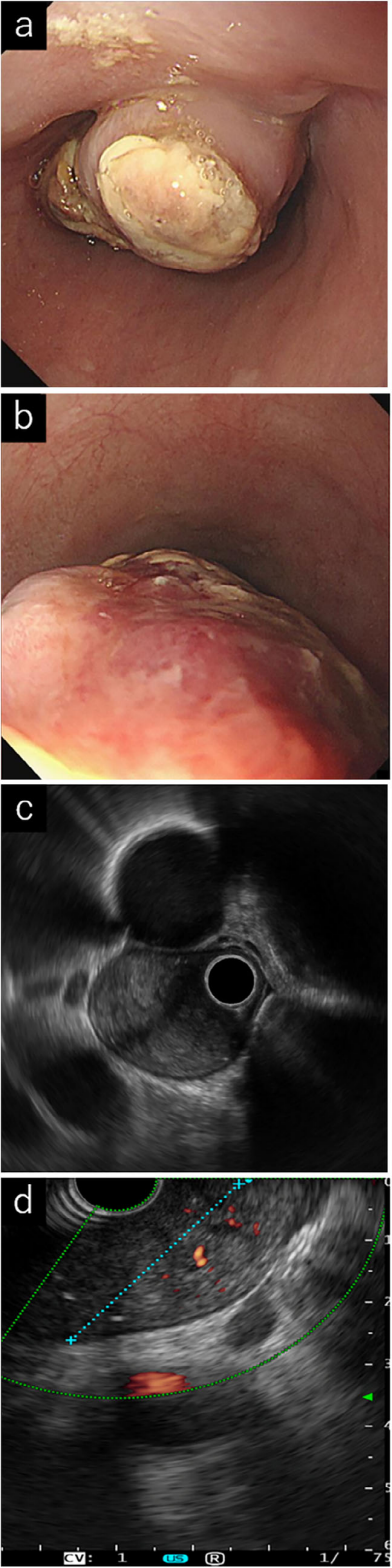
Endoscopic and endoscopic ultrasound findings after tumor enlargement to 5 cm. (a) Tumor with surface ulceration and a white coating, observed 2 years before endoscopic resection. (b) Reddening on the anal side of the tumor. (c) Endoscopic ultrasound image showing an intact muscle layer. (d) Doppler imaging demonstrating high echogenicity, suggesting internal bleeding and abundant blood flow.

One year before ESD, the patient developed worsening dysphagia. EGD at that time revealed tumor enlargement to 5 cm with an erythematous and ulcerated apex covered with white exudates (Figure 2a,b). Over the preceding years, nine endoscopic examinations and 62 biopsies failed to yield a definitive diagnosis; a biopsy of an exposed tumor margin showed only exudate and granulation tissue. Endoscopic ultrasound (EUS) demonstrated an oval lesion with heterogeneous echogenicity arising from the third layer, while the fourth layer remained intact; high‐echogenicity areas suggested internal hemorrhage, and Doppler imaging confirmed abundant blood flow (Figure 2c,d). Fine‐needle aspiration (FNA) with a 22‐gauge needle yielded peripheral fibrous tissue, granulation tissue, and debris, but no epithelial or neoplastic cells.

Contrast‐enhanced chest computed tomography (CT) showed a mosaic‐like high‐attenuation esophageal lesion without lymphadenopathy, and positron emission tomography‐CT revealed no abnormal uptake (Figure S1). Despite these inconclusive findings, we proceeded with ESD for both diagnostic and therapeutic purposes, given the tumor's rapid growth and the patient's worsening dysphagia.

Given the tumor's large size and lack of a definitive diagnosis, ESD was performed under general anesthesia to mitigate the risk of unexpected complications. A submucosal injection of a 1:1 mixture of hyaluronic acid and glycerol with indigo carmine was administered at the base of the semi‐pedunculated lesion, achieving excellent lift. After making appropriate circumferential incisions, submucosal dissection was advanced from the oral to anal side with a needle knife. The submucosal layer was well visualized without significant fibrosis, although abundant submucosal vessels and frequent minor bleeding were noted. En bloc resection was accomplished without severe bleeding or perforation, and the specimen was retrieved orally with a snare (Video S1).

**FIGURE 3:**
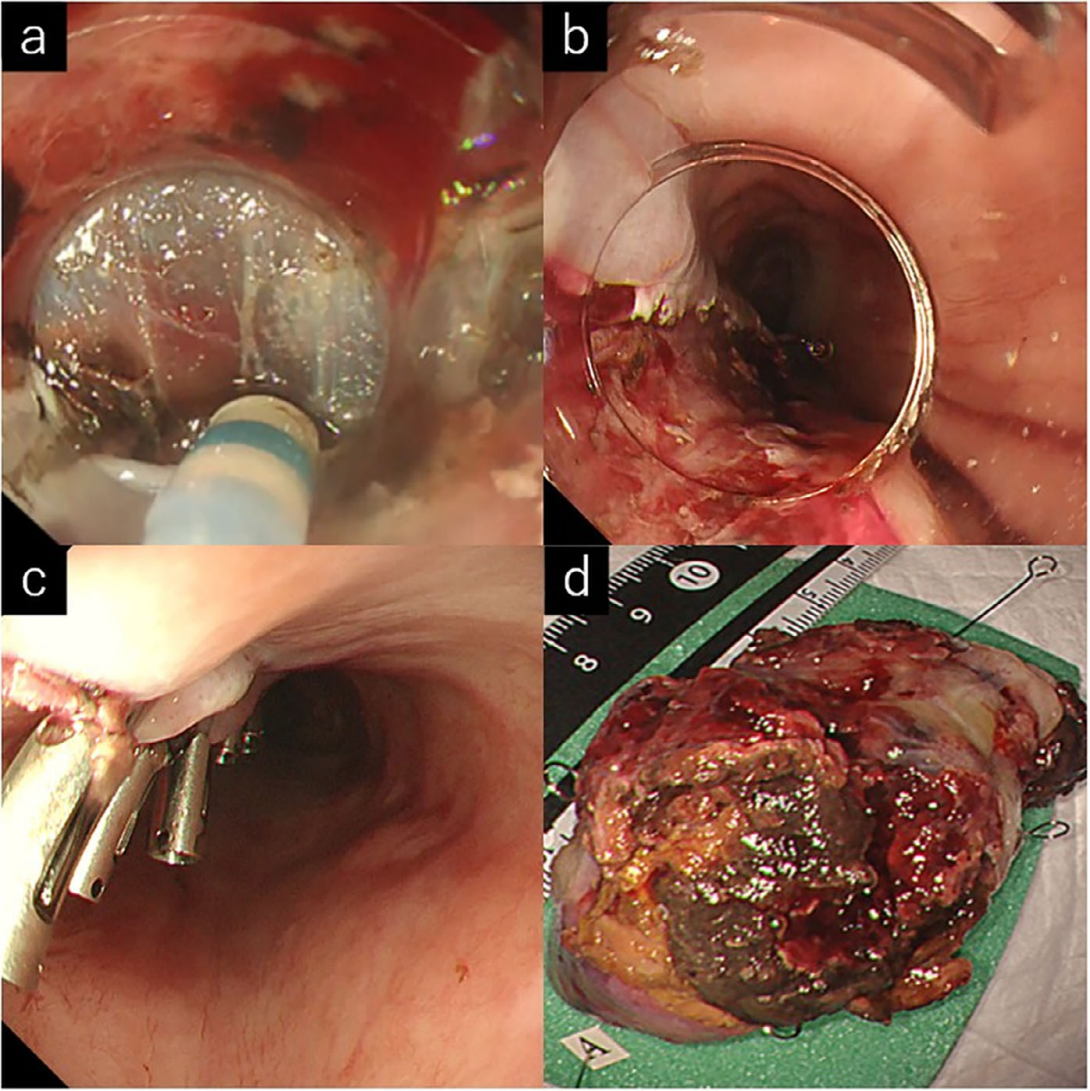
Endoscopic images during endoscopic submucosal dissection (ESD). (a) Submucosal dissection using a needle knife. (b) Longitudinal mucosal defect immediately after ESD. (c) Complete closure of the defect with endoscopic clips. (d) Resected specimen demonstrating successful en bloc removal of the > 50 mm lesion.

The esophageal surface appeared ulcerated, and the tumor tissue was friable. The tumor was dissected relatively easily because it was semi‐pedunculated and showed no fibrosis. The tumor base was small, allowing closure of the defect after ESD using clips (Figure 3c). The procedure was completed in 30 min with minimal intraoperative bleeding and no complications. The patient was discharged on postoperative day six. The patient's postoperative course was uneventful, with significant improvements in dysphagia. EGD and CT performed at the 6‐month follow‐up showed no recurrence (Figure S2).

**FIGURE 4:**
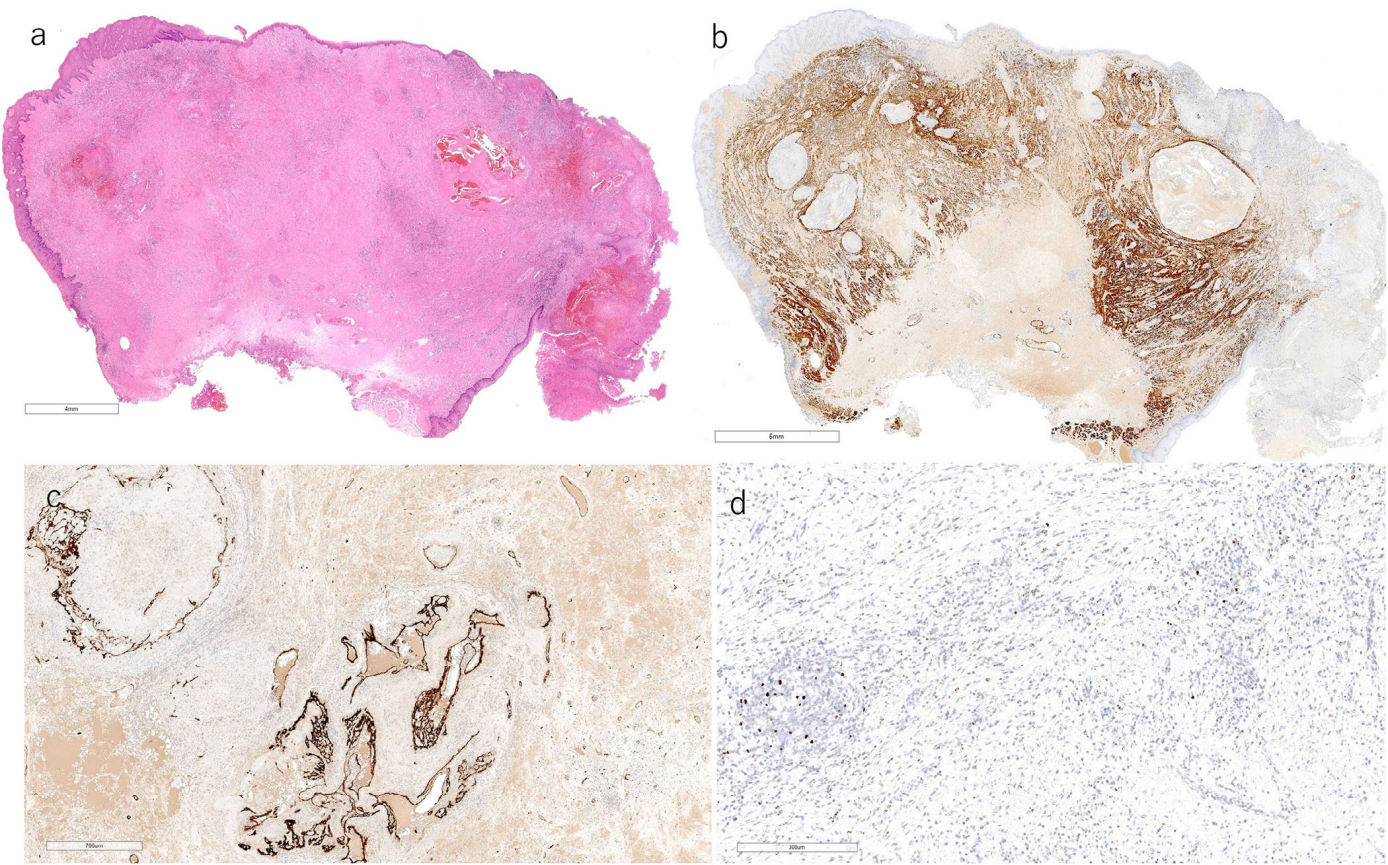
Histological findings: (a) Hematoxylin and eosin (HE) staining. An elevated lesion with surface ulceration. Dilated capillaries of various sizes and organized thrombus with recanalization. Hemorrhage, edema, and inflammatory cell infiltration were present between smooth muscle bundles. Dilated capillaries of various sizes and organized thrombus with recanalization. (b) Desmin immunohistochemistry (IHC), corresponding to (a). Markedly thickened muscularis mucosae. (c) CD34 IHC, corresponding to CD34 IHC, corresponding to (a). Endothelial cells were highlighted. (d) Epstein‐Barr Virus encoded RNA in situ hybridization (EBER‐ISH). A small number of lymphocytes were positive for EBER‐ISH (the photograph shows a hot spot). Scale bars: a = 4 mm; b = 700 µm; c = 5 µm; d = 300 µm.

The histopathological examination revealed an elevated lesion with superficial ulceration (Figure 4a). This increase was mainly due to the expansion of the muscularis mucosae and submucosal layers. The muscularis mucosae was markedly thickened, and its smooth muscle showed a disorganized arrangement (Figure 4b). However, no distinct masses were identified, ruling out neoplastic lesions. The proliferation of dilated capillaries of various sizes was prominent between the smooth muscle bundles and was accompanied by hemorrhage and inflammatory changes, including edema, fibrosis, and inflammatory cell infiltration, mainly lymphocytes (Figure 4a). An organized thrombus with recanalization was observed within the vessels (Figure 4a,c). Only a few infiltrating lymphocytes were positive for EBV‐encoded RNA in situ hybridization (EBER‐ISH) (Figure 4d).

## 2 Discussion

This case involved an esophageal SEL that remained stable for several years before rapidly enlarging and causing dysphagia. Despite multiple preoperative biopsies and FNA, a definitive diagnosis had not been established. The final pathological examination revealed vascular proliferation, inflammatory changes, and mucosal thickening without distinct mass formation, making classification under any established pathology difficult. A small number of EBER‐ISH‐positive cells were observed in the biopsy specimens, but the findings did not match classical EBV‐associated conditions. However, the possibility of an EBV‐associated lesion cannot be ruled out. The patient had undergone tracheostomy for laryngeal edema and had a history of recurrent exudative otitis media, sinusitis, and pneumonia. However, the absence of typical features of ANCA‐related vasculitis or rheumatoid disease complicated the diagnosis. Notably, inflammation and tissue changes may have been exacerbated by repeated biopsies (62 biopsies). Based on the timing and frequency of multiple biopsies (a total of nine endoscopic examinations with biopsies over the last year),

We considered differential diagnoses. Leiomyoma, the most common benign submucosal esophageal tumor a usually arises from the muscularis propria in the middle to lower esophagus as a well‐demarcated mass with an intact mucosa [4], but our lesion lacked clear mass formation and instead showed prominent vascular proliferation, atypical for leiomyomas of the muscularis mucosae. Fibrovascular polyp, which may evolve without adipose tissue and typically appears in the cervical esophagus as a fibrovascular stalk covered by squamous epithelium, was unlikely because our lesion was in the thoracic esophagus, lacked a polypoid stalk or pedunculated morphology both endoscopically and histologically (despite over 60 biopsies and the presence of ulceration and granulation tissue), and instead featured thickened mucosa, abundant dilated capillaries, hemorrhage, and chronic inflammatory changes. [5]. IgG4‐related disease requires (1) diffuse or localized swelling /masses, (2) serum IgG4≥135 mg/dL, and (3) histopathological findings showing 40% IgG4+/IgG+ plasma cells, and >10 IgG4+cells per high‐power field with marked lymphoplasmacytic infiltration and fibrosis. —but our specimen had scarce IgG4(< 10% ratio)‐ minimal plasma‐cell infiltration, and no significant fibrosis. [6] Finally, Dieulafoy's lesion, characterized by a small (1–3 mm) submucosal arterial malformation that can elevate over time, was ruled out as no such arterial defect was seen, suggesting instead hemorrhagic capillary or venous origin [7].

To the best of our knowledge, there have been no previous reports of rapidly enlarging esophageal SELs that remained histopathologically unclassified even after resection and were successfully treated with ESD. No recurrence was observed during the subsequent 15 months of follow‐up after ESD.

Appropriate preoperative endoscopic localization of the tumor origin using EUS enabled the large, space‐occupying lesion to be safely and completely excised via a minimally invasive approach. His case highlights key considerations in managing esophageal SELs. Although most SELs are benign and remain stable for years, clinicians should recognize that atypical growth patterns, such as rapid enlargement or symptomatic progression, can occur after prolonged quiescence. Careful, continuous monitoring of even initially indolent‐appearing lesions may therefore be warranted. Preoperative EUS is crucial for accurately identifying the lesion's layer of origin and the extent of surrounding involvement, guiding the choice of intervention, such as ESD. Furthermore, repeated tissue sampling may provoke inflammatory or reactive changes, potentially altering the lesion's presentation. ESD can serve not only as a minimally invasive curative procedure but also as a definitive diagnostic strategy when malignancy cannot be excluded by preoperative evaluation alone. This case underscores the diagnostic challenges of SELs and illustrates ESD's dual role as both a therapy and a diagnostic modality in selected patients.

## Author Contributions

**Mai Fukuda**: review and editing (equal); **Naoya Tada**: review and editing (equal); **Koichi Oishi**: review and editing (equal); **Mamoru Ito**: review and editing (equal); **Yuko Hasegawa**: review and editing (equal); **Toshiki Futakuchi**: review and editing (equal); **Masakuni Kobayashi**: review and editing (equal); **Naoto Tamai**: review and editing (equal); **Miku Maeda**: review and editing (equal); **Masayuki Shimoda**: review and editing (equal); **Kazuki Sumiyama**: review and editing (equal).

## Conflicts of Interest

Kazuki Sumiyama is a deputy editor‐in‐chief of DEN Open. The rest of the authors declare no conflicts of interest.

## Ethics Statement

N/A

## Supporting information




**FIGURE S1** Contrast‐enhanced chest CT revealed a mass in the thoracic esophagus with a mosaic‐patterned high‐attenuation area.

**FIGURE S2** Post‐ESD scar at 8‐month follow‐up, showing no evidence of recurrence.

**VIDEO S1** The video demonstrates an ESD procedure for a large semi‐pedunculated esophageal lesion.

## References

[1] T. Nishida , N. Kawai , S. Yamaguchi , and Y. Nishida , “Submucosal Tumors: Comprehensive Guide for the Diagnosis and Therapy of Gastrointestinal Submucosal Tumors,” Digestive Endoscopy 25 (2013): 479–489. 23902569 10.1111/den.12149

[2] M. A. Schneider , D. Vetter , and C. A. Gutschow , “Management of Subepithelial Esophageal Tumors,” Innovative Surgical Sciences 10 (2025): 21–30. 40144787 10.1515/iss-2023-0011 PMC11934943

[3] C. Verloop , L. Hol , M. Bruno , L. Van Driel , and A. D. Koch , “Endoscopic Resection in Subepithelial Lesions of the Upper Gastrointestinal Tract: Experience at a Tertiary Referral Hospital in The Netherlands,” Endoscopy International Open 12 (2024): E868–E874. 38989251 10.1055/a-2325-3747 PMC11236476

[4] W. Jiang and T. W. Rice , “Esophageal Leiomyoma: Experience From a Single Institution,” Diseases of the Esophagus 26 (2013): 167–174. 22458777 10.1111/j.1442-2050.2012.01345.x

[5] R. Lüthen , U. Janzik , R. Derichs , H. Balló , and U. Ramp , “Giant Fibrovascular Polyp of the Esophagus,” European Journal of Gastroenterology & Hepatology 18 (2006): 1005–1009. 16894315 10.1097/01.meg.0000228971.34280.57

[6] H. Umehara , K. Okazaki , and H. Ohara , “Comprehensive Diagnostic Criteria for IgG4‐related Disease (IgG4‐RD), 2011,” Modern Rheumatology 22 (2012): 21–30. 22218969 10.1007/s10165-011-0571-z

[7] R. Chaer and W. S. Helton , “Dieulafoy's Disease,” American Journal of Surgery 196 (2003): 290–296. 10.1016/S1072-7515(02)01801-X 12595057

