# Supplementary figures and images for: Successful Endoscopic Excision for a Rapidly Enlarging Esophageal Histopathologically Unclassified Subepithelial Lesion: A Case Report
Source: DEN Open. 2025 Oct 15;6(1):e70219. doi: 10.1002/deo2.70219 (PMC12527641; doi:10.1002/deo2.70219)

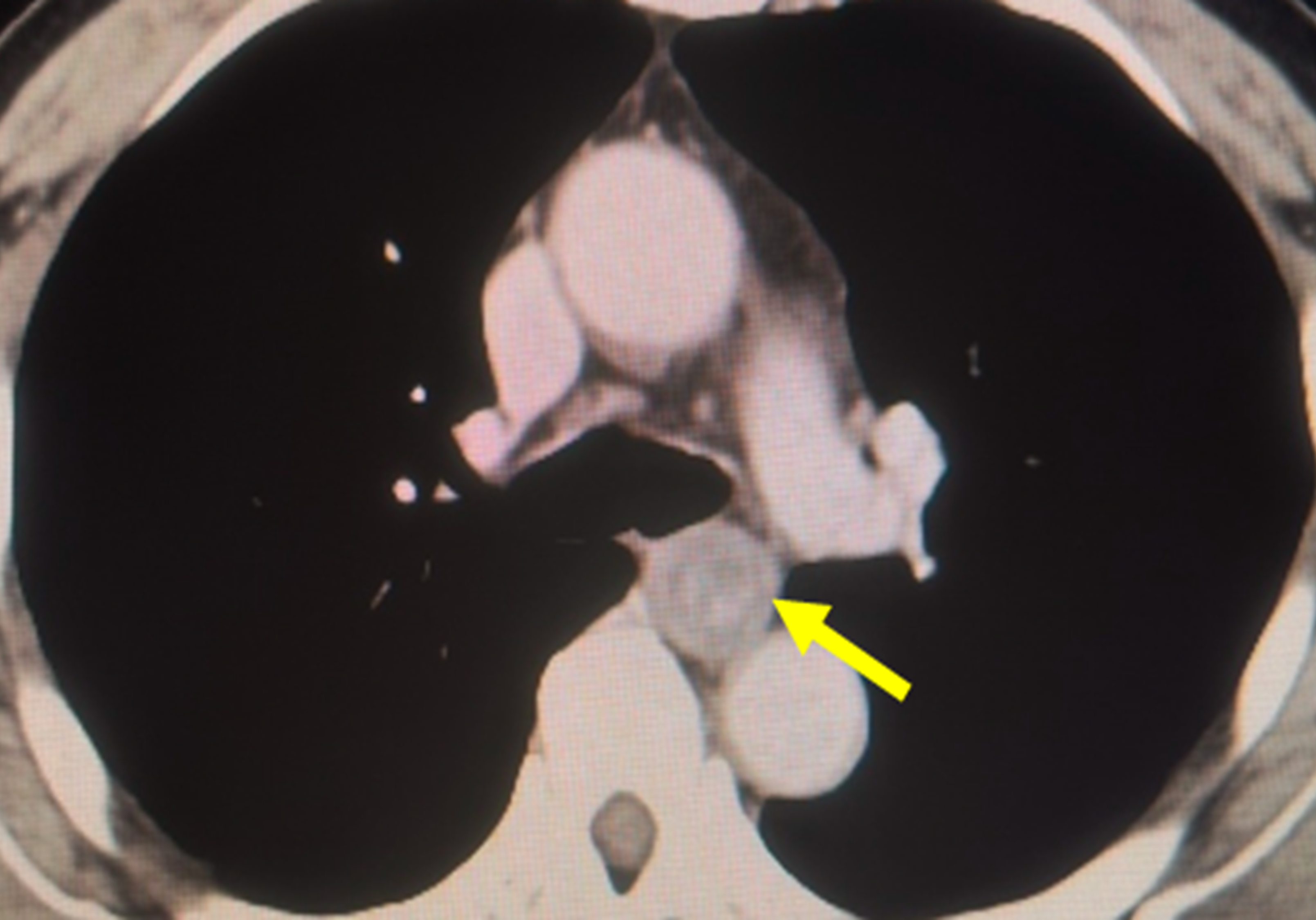

Supplement: Supplementary file 1 — FIGURE S1 Contrast‐enhanced chest CT revealed a mass in the thoracic esophagus with a mosaic‐patterned high‐attenuation area. [file DEO2-6-e70219-s001.TIF]

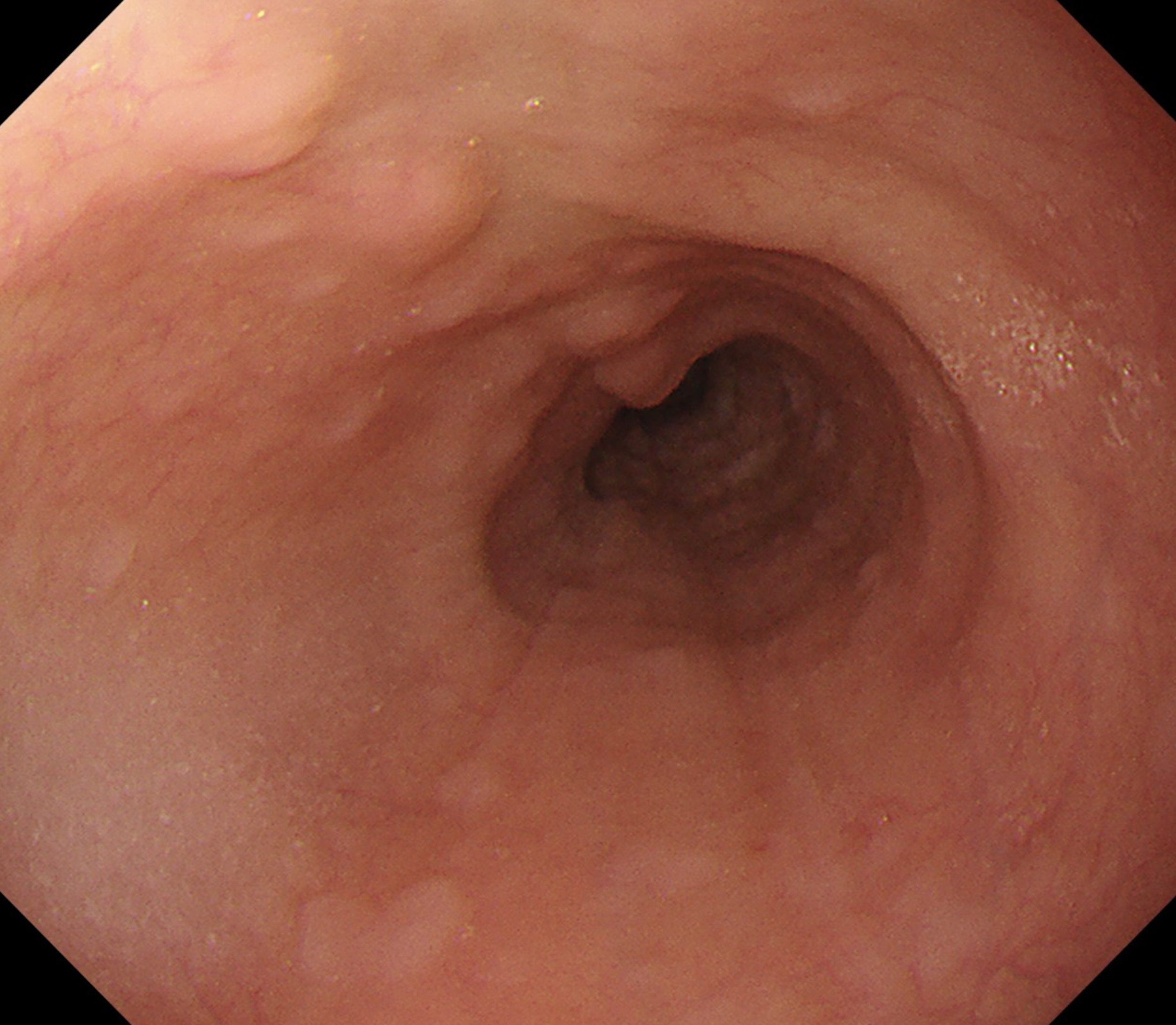

Supplement: Supplementary file 2 — FIGURE S2 Post‐ESD scar at 8‐month follow‐up, showing no evidence of recurrence. [file DEO2-6-e70219-s003.tif]
