# Supplementary material for: Successful Endoscopic Excision for a Rapidly Enlarging Esophageal Histopathologically Unclassified Subepithelial Lesion: A Case Report
Source: DEN Open. 2025 Oct 15;6(1):e70219. doi: 10.1002/deo2.70219 (PMC12527641; doi:10.1002/deo2.70219)
Supplement: Supplementary file 3 — VIDEO S1 The video demonstrates an ESD procedure for a large semi‐pedunculated esophageal lesion. [file DEO2-6-e70219-s002.docx]

Video can be viewed from here:

[DENOP-2025-0108.R1 video](https://wiley-my.sharepoint.com/:f:/p/yikegami/ErRzEuvvLcRCp4Nk76QWgH8B7g76tancBI-uE8_nLNH7mQ?e=WhxlPi)
